# Genomic epidemiology of Salmonella Enteritidis human infections in the Netherlands, 2019 to 2023

**DOI:** 10.1099/mgen.0.001394

**Published:** 2025-04-23

**Authors:** Linda E. Chanamé Pinedo, Eelco Franz, Timothy J. Dallman, Claudia E. Coipan, Roxanne Wolthuis, Kees T. Veldman, Lapo Mughini-Gras, Roan Pijnacker, Maaike J.C. van den Beld

**Affiliations:** 1 Centre for Infectious Disease Control, National Institute for Public Health and the Environment (RIVM), Bilthoven, Netherlands; 2 Institute for Risk Assessment Sciences, Utrecht University, Utrecht, Netherlands; 3 Wageningen Bioveterinary Research (WBVR), part of Wageningen University and Research, Lelystad, Netherlands

**Keywords:** antimicrobial resistance, epidemiology, genome, *Salmonella enterica*

## Abstract

*Salmonella enterica* serotype Enteritidis (SE) is a common foodborne pathogen that can cause human salmonellosis. Identifying closely related cases is essential to control the pathogen through, e.g. outbreak investigation, but it is often challenging due to the low genetic diversity of SE, particularly with traditional typing methods. This study aimed to investigate the population structure of SE genomes collected during routine surveillance in the Netherlands using whole-genome sequencing (WGS), their clustering, temporal distribution and the association between epidemiological and phenotypic antimicrobial resistance (AMR) factors and the persistence of SE clusters. We also investigated the distribution of genotypic AMR markers among these isolates. The study collection comprised 1,669 unique SE isolates from human infections collected from Dutch surveillance between 2019 and 2023, and their relatedness was derived using core-genome multi-locus sequence typing and Hamming distances. Based on the results, the 216 clusters comprised 1,085 sequences, in addition to 584 sequences depicted as singletons. These clusters predominantly fell within three major lineages, of which two were the previously described Global and Atlantic lineages. Of these clusters, approximately a third persisted for more than 1 year during the 5-year study period. However, no statistically significant associations were found between epidemiological factors, such as age, gender and travel history, or phenotypic AMR and the persistence of SE clusters. The most common AMR genetic markers observed were related to antimicrobial classes of (fluor)quinolones, *β*-lactamases and aminoglycosides. This study provides a better understanding of the genomic epidemiology of SE in the Netherlands based on WGS. Further analysis that includes samples from the food-chain supply, along with higher resolution methods during a post-Coronavirus Disease of 2019 (COVID-19) period, may provide more insights into the possible causes of the persistence of SE clusters.

## Data Summary

Raw sequence data, or assemblies when raw reads were not available, were submitted to the European Nucleotide Archive, study numbers: PRJEB54672 (sequences from January 2019 to March 2020) and PRJEB60024 (sequences from April 2020 to December 2023). The authors confirm that all supporting data have been provided within the article or through supplementary data files.

**Impact Statement**

Whole-genome sequencing has become a routine tool for typing foodborne pathogens like *Salmonella enterica* serotype Enteritidis (SE) in the Netherlands since 2019. This has greatly improved the characterization of closely related isolates for surveillance and outbreak investigation purposes. On a pragmatic surveillance level, our study showed that a cut-off of ≤3 alleles is optimal for clustering of SE isolates using core-genome multi-locus sequence typing. The present study also revealed an extensive degree of clustering with SE isolates, presenting challenges in public health surveillance. Consequently, a triage system is recommended to prioritize source tracing for specific clusters based on factors such as growth rate, age, symptomatology and distribution. Furthermore, our findings indicate that approximately one-third of the clusters were temporally persistent (lasting more than 1 year). This persistence, which was previously observed primarily in *Listeria monocytogenes* due to its ability to endure in food production environments and some prior re-emerging SE outbreaks across the EU, now appears to have a significant role in the epidemiology of SE as well. This new insight highlights the importance of focusing investigative efforts on persistent SE clusters, which account for around 22% of cases. By targeting these persistent clusters, the likelihood of identifying sources increases, potentially leading to a greater reduction in disease burden.

## Introduction

*Salmonella* is the second most prevalent zoonotic pathogen in Europe [1]. Among more than 2,500 serotypes, *Salmonella enterica* subspecies *enterica* serotype Enteritidis (SE) is the most common, contributing significantly to foodborne outbreaks across European countries [1]. Notably, in the Netherlands, SE alone accounts for 28% of all salmonellosis cases [2]. After a sustained decline until 2015, SE incidence showed a resurgence in recent years [3]. Additionally, despite the impact of the Coronavirus Disease of 2019 (COVID-19) measures, which resulted in the lowest incidence ever recorded in the Netherlands, SE is currently on the rebound to pre-pandemic levels [4]. SE is predominantly associated with poultry, especially laying hens, with eggs serving as a primary source of human infection [5].

In recent years, public health authorities across the European Union (EU) have made substantial efforts in adopting whole-genome sequencing (WGS) for surveillance purposes and outbreak investigations [6]. In the Netherlands, the use of WGS and core-genome multi-locus sequence typing (cgMLST) has been incorporated as routine typing methods for outbreak investigation and the surveillance of SE human infections since 2019 [7]. Due to the high resolution of the typing, WGS has enhanced the ability to detect clusters/outbreaks and to identify their sources with more certainty, especially for more frequently encountered *Salmonella* serotypes, such as SE [8]. This is crucial because multiple clusters of SE isolates can appear simultaneously and spread across different areas. These clusters would not have been distinguished by traditional typing methods such as serotyping or molecular methods such as pulsed-field gel electrophoresis (PFGE) and multiple-locus variable-number tandem repeat analysis (MLVA) [9,10].

Several multi-country outbreaks of SE across the EU have demonstrated the potential for the re-emergence of outbreak-related strains in the food chain over several years [6,9]. For instance, one of the largest multi-country outbreaks of SE in Europe linked to contaminated eggs from Poland in 2016 saw a rise in cases towards the end of 2017, despite control efforts [6]. Evidence suggests that the outbreak even continued until at least 2019 [11]. The exact source of the persistent clusters remained unidentified. This persistence suggests that clusters can be repeatedly introduced in the food chain despite control measures. Quantifying the occurrence and characterization of these persistent clusters, as well as the potential factors associated with their persistence, may provide more insights into source tracing and control options.

Antimicrobial resistance (AMR) is an ongoing public health issue affecting the treatment and control of *S. enterica* infections in both humans and animals. The emergence of antimicrobial-resistant *S. enterica* strains in livestock poses a significant risk to human health, as these strains can spread from livestock reservoirs to humans [12]. Additionally, the survival of *S. enterica* in animal reservoirs can be partly attributed to AMR, leading to an increased risk of human exposure to the pathogen. Over the past decade, the Netherlands has seen a gradual increase in the prevalence of AMR in SE isolates causing human infections, particularly for those resistant to (fluor)quinolones, ampicillin and sulfamethoxazole [13]. Besides the clinical consequences of AMR, it is essential to gain more understanding of the role of AMR in the epidemiology of SE clusters. We hypothesized that AMR could contribute to the temporal persistence/re-emergence of clusters due to the potential for AMR to enhance the persistence of pathogens at the farm level and its specific role in the sustained presence of these clusters over time.

This study aimed to characterize the genomic epidemiology of SE using WGS data of isolates from human infections between 2019 and 2023. Specifically, the study investigated the extent of SE clustering and its temporal distribution, while also examining factors associated with the persistence of clusters such as epidemiological factors and phenotypic AMR. Furthermore, we described the distribution of antimicrobial and metal-biocide resistance gene markers present among these SE isolates, using WGS.

## Methods

### Data collection and study population

Culture-confirmed *Salmonella* isolates from human infections reported in the Netherlands between January 2019 and December 2023 were obtained from the Dutch National Surveillance System for *Salmonella*. These isolates were collected from a network of clinical microbiology laboratories across all the Netherlands, which voluntarily sent *Salmonella* isolates obtained from their routine diagnostic activities among patients with salmonellosis-compatible symptoms, along with a minimal set of patient metadata (see below), to the Dutch National Institute for Public Health and the Environment (RIVM) for further typing and characterization [14]. From 2019 to 2020, *Salmonella* serotyping was performed using a pre-screening with the Luminex technique (xMAP *Salmonella* Serotyping Assay kit), followed by confirmative classical slide agglutination with *Salmonella* O- and H-antisera according to the White–Kauffmann–Le Minor scheme [15]. From 2021 onwards, serotyping was exclusively conducted via *in silico* methods, facilitated by an in-house developed pipeline grounded on SeqSero2, using micro-assembly mode [16]. Only isolates that were serotyped as SE and of which the quality of the WGS sequences passed our quality criteria were included in this study. Metadata included sampling date, age, sex, specimen type (faeces, blood, urine etc.) and travel history where available. Phenotypic antimicrobial susceptibility testing (AST) of *Salmonella* isolates was performed on a random sample of isolates based on criteria that selects ~65% of isolates according to source, laboratory and serotype [14]. Broth microdilution was employed to obtain the minimum inhibitory concentration (MIC) values according to the European Committee on Antimicrobial Susceptibility Testing (EUCAST) guidelines [17]. The MIC values were then used to classify the isolates as resistant (non-WT) or susceptible (WT) based on the epidemiological cut-offs (ECOFFs) set by EUCAST for *Salmonella enterica*. The detailed ECOFF values used in this study can be found in Table S1 (available in the online Supplementary Material).

### Case definition and unit of analysis

A case was defined as a patient with a culture-confirmed *Salmonella enterica* infection caused by SE. If more than one SE isolate was collected from the same case, only the first isolate was included. The isolates, which underwent WGS, and the resulting raw reads that were assembled into genomic sequences were referred to as sequences. Cases served as the primary unit of analysis for epidemiological analysis, while sequences were employed as the primary unit for genomic analyses.

### Epidemiological factors (metadata) and phenotypic AMR

Age was summarized using median and interquartile range (IQR), and sex was categorized as ‘female’, ‘male’ or unknown. The type of infection was defined as ‘non-invasive’ if isolates were cultured from faeces, urine, vomit, sputum, skin, soft tissue or abscesses and as ‘invasive’ if isolates were cultured from blood, cerebrospinal fluid, peritoneal fluid, pleural fluid, synovial fluid, bone or other normally sterile sites [2]. Travel history (meaning outside of the Netherlands) was categorized as ‘yes’ and ‘unknown’. Because filling in travel history by the submitter is not mandatory and negative reporting is not possible, isolates without information on travel history were categorized as ‘unknown’ travel history. Phenotypic AMR variables only included antimicrobials with a minimum of five resistant counts per year. This criterion was used to ensure greater accuracy in the estimates from the regression model in the epidemiological analysis. ‘Resistant counts per year’ refers to the annual count of instances where resistance was observed among SE isolates. These antimicrobials comprised ampicillin, (fluor)quinolones (ciprofloxacin and nalidixic acid) and sulfamethoxazole. Subsequently, the annual resistance proportion for each of these antimicrobials was calculated as the total number of SE isolates resistant to a specific antimicrobial divided by the total number of SE isolates tested for phenotypic AST.

### DNA extraction, WGS and sequence analysis

WGS has been routinely performed on SE isolates for national surveillance and outbreak investigation purposes in the Netherlands since 2019. DNA extraction was performed using either the GenElute Bacterial Genomic DNA kit (Sigma-Aldrich, Inc.) or automated with the Maxwell® RSC Instrument using the cultured cells DNA kit (Promega Corp.), both according to the manufacturers' protocol. Library preparation was performed using the Illumina Nextera XT DNA Library Preparation kit or the Illumina DNA prep kit. Libraries were run on the Illumina NextSeq 500 or 550 machine, resulting in 2×150 bp reads.

The reads were processed through an in-house developed pipeline named ‘Juno assembly’ v3.0.3, with default parameters [18]. This involved read trimming, extensive quality control checks of raw reads, trimmed reads and assemblies, followed by *de novo* assembly. Raw reads with a Phred quality score >30 and resulting *de novo* assemblies with a total length between 4.4 and 5.8 Mbp, *N*_50_ >30,000 bp, GC% of 51.6–52.3%, number of contigs <300, average coverage ≥30×, genome completeness >96% and contamination <4% were considered as good quality and were used for analysis. The pipeline employed the following tools and versions: FASTQC v0.11.9, FastP v0.20.1, Picard v2.26.0, SPAdes v3.15.3, QUAST v5.0.2, CheckM v1.1.3, Bbtools (bbmap) v38.86, MultiQC v1.11, Kraken2 v2.1.2 and Bracken 2.6.1. Using the output of the Juno assembly pipeline, an in-house developed pipeline named ‘Juno typing’ v0.7.1 based on SeqSero2v1.1.1 [16,19] was applied for *in silico* serotyping and 7-locus multilocus sequence typing (MLST) of *Salmonella* sequences. *De novo* assemblies were imported into Ridom SeqSphere v9.0.8, where allelic profiles were determined using the Enterobase *Salmonella enterica* v2.0 cgMLST scheme comprising 3,002 loci [20]. To determine whether sequences belong to the previously described Global (sequences from all inhabitants worldwide) or Atlantic lineages (sequences predominantly from the USA and Europe), as well as other lineages present in this study, sequences were compared phylogenetically against representative isolates from [21]. Specifically, 449 non-redundant genomes from Li *et al*. were aligned to the SE reference genome P125109 (accession: AM933172) using Snippy v4.6.0. SNP positions of high quality defined by a mapping quality >30, depth >10 and variant ratio >0.9 were imported into SnapperDB v0.2.5 [22]. Hierarchical single linkage clustering was performed on the pairwise SNP difference between all isolates at descending distance thresholds. The Δ250 SNP cluster threshold corresponded to the lineage delineations of Li *et al.* Random representatives from each Δ250 cluster were incorporated into this study set, and the lineage of the Dutch isolates was assigned based on monophyletic co-clustering.

The detection of genes related to AMR, as well as resistance to metals and biocides, was conducted using an in-house-developed pipeline named ‘Juno-AMR’ v0.8 [23]. This pipeline is based on ResFinder (including pointfinder) v4.4.1 [24] for AMR gene detection and AMRFinderPlus v3.12.8 [25] for identification of metal and biocide resistance genes, as well as AMR genes. For Resfinder, optional parameters were set to minimum coverage of 0.6 and an identity threshold of 0.8. In the case of AMRFinderPlus, the ‘--plus’ flag was used. Isolates were considered potentially multidrug-resistant if genetic AMR markers predicted resistance against ≥3 separate classes of antimicrobials.

### Phylogenetic and cluster analysis using cgMLST

Using the allelic profiles output from SeqSphere, the pairwise distance between sequences was measured as Hamming distances, using all cgMLST loci and ignoring pairwise missing loci. Hierarchical clustering was defined from the distance matrix using the three most commonly used linkage criteria [26]: single, average and complete, using the ‘hclust’ function in the R package stats v. 4.3.2 [27]. The fit of clustering to the distance matrix was assessed using the cophenetic correlation coefficient [28] (‘cophenetic’ function from the same stats package) for each of the three clustering algorithms. The cophenetic correlation coefficient value ranges between 0 and 1, with a value of 1 indicating a perfect fit between the dendrogram and the distance matrices. The linkage with the highest value was selected for tree topology [26].

The optimal number of clusters was established on the consensus of three internal validity indices: silhouette [29], McClain-Rao [30] and Dunn2 index [31] as described in [26]. In brief, the silhouette index evaluates the effectiveness of data clustering by calculating the average silhouette score for each partition, which is the mean of the silhouette values for all objects in the dataset. The silhouette value for an individual object is defined as the difference between the mean intra-cluster distance (the average distance of the object to all other objects within the same cluster) and the smallest mean inter-cluster distance (the average distance of the object to all objects in the nearest different cluster). The McClain–Rao index is calculated as the ratio of the mean intra-cluster distance to the mean inter-cluster distance. The Dunn2 index evaluates clustering by measuring the ratio of the minimum average intra-cluster dissimilarity to the maximum inter-cluster dissimilarity. These indices were estimated using the functions from ‘Nbclust’ and ‘cluster.stats’ from the R packages Nbclust v. 3.0.1 [32] and fpc v. 2.2–11 [33]. Since the estimation of clustering of SE for outbreak investigation in the Netherlands and Europe currently uses five allelic distances in the national cgMLST [9], a range of allelic cut-offs for the definition of clusters from *k*=2 to *k*=5 was evaluated for internal validation. An optimal cluster definition (and later used for cluster analysis) was considered as having Dunn2 and silhouette indices at highest values and the McClain–Rao at lowest values. The resulting clusters were labelled with the initial of the linkage method, followed by an integer, ranging from one to the total number of clusters identified using each linkage method. Finally, a dendrogram was estimated based on the best-fitted hierarchical clustering. To enhance visualization, this dendrogram was pruned by randomly selecting one member from each cluster.

The travel-related SE cases might be underestimated by the surveillance system. To infer whether a cluster was likely to be travel-related, we compared the observed number of travel-related cases within a cluster to the expected number of travel-related cases, assuming a beta-binomial distribution. The shape parameters for this distribution were defined by the total number of SE cases eligible for WGS, denoted as *n*, and the number of travel-related cases, denoted as *j*. These parameters are expressed as s_1_=*j* and s_2_=*n*–*j*. For each cluster size and all possible numbers of travel-related cases therein, we calculated the probability of finding a number of travel-related cases higher or equal to the observed number of travel cases using a cumulative beta binomial distribution function (CDF), defined as one minus the CDF for the observed number of travel cases. This probability is defined as *P*(*X*≥*x*)=1−CDF(*x*,cluster_size_,s_1_,s_2_). Additionally, we estimated the expected number of cases in a cluster by simulating 10,000 random samples from a beta-binomial distribution, using the cluster size and the predefined shape parameters, and then calculating the mean of these samples. This mean value is subsequently rounded to the nearest whole number to facilitate interpretation. This can be expressed as, $E\;(T)=round\left(\frac{1}{N}\sum_{i=1}^{N}BB\left(x,s_{1},s_{2}\right)i\right)$; where *N=10,000* is the number of simulations; $BB{\left(x,s_{1},s_{2}\right)}_{i}$ is the *i*th random draw with the parameters *x*, $\frac{1}{N}\sum_{i=1}^{N}BB{\left(x,s_{1},s_{2}\right)}_{i}$ is the sum of all simulated values and $\frac{1}{N}\sum_{i=1}^{N}BB{\left(x,s_{1},s_{2}\right)}_{i}$ is the mean of the simulated values. The results of this calculation can be found in Table S2. Finally, if the number of travel-related cases within a cluster was found to be higher than expected by chance, the cluster was considered entirely travel-related.

Clusters that were present for over a year were considered ‘persistent’, regardless of continuity. Those present for less than a year were considered ‘non-persistent’. Clusters that were observed for the first time less than 1 year before the end of the study period were labelled as ‘unknown persistent’, as their persistence beyond a year could not be observed. For sensitivity analysis, any differences in the number of persistent clusters identified by the aforementioned hierarchical clustering methods were estimated.

### Factors associated with persistence of clusters

To assess whether epidemiological factors (age, gender, type of infection and travel history) and phenotypic AMR to ampicillin, (fluor)quinolones and sulfamethoxazole of SE cases (those eligible for WGS) were associated with persistence of clusters, mixed-effects Cox proportional hazard regression analysis was conducted. The analysis was performed using the coxme package in R (version 2.2–18.1) [34] and included cluster as a random intercept to account for the correlation between cases within clusters. The model allowed us to account for the timing of the first occurrence of cases within each cluster. Cases from clusters with unknown persistence were treated as censored in the response variable.

Initially, associations were assessed by using univariable analysis. Subsequently, if significant associations (*P*<0.05) were observed, these were then selected for multivariable regression. The ‘cox.zph’ function from the R survival package version 3.5–7 [35] was used to assess the proportional hazard assumption, which posits that the effect of predictor variables on the hazard rate remains constant over time. Hazard ratios (HRs) and their 95% confidence intervals (CIs) and *P* values were obtained from each model.

All phylogenetic and cluster analyses, annotation and epidemiological analyses were conducted using R software version 4.3.3 [27].

## Results

### Description of study population

From January 2019 to December 2023, 5,802 *Salmonella* isolates from human cases were retrieved from the Dutch surveillance system. After removing 704 repeated isolates from the same patients, a total of 5,098 isolates were selected. Among these *Salmonella* isolates from unique patients, 1,677 (32.9%) were phenotypically serotyped as SE and/or confirmed as SE using *in silico* determination with the Juno typing pipeline [16]. Of the 1,677 isolates, 598 were serotyped as SE using traditional phenotypical methods (antiserum), and 1,079 were confirmed as SE using molecular methods with the *in silico* Juno typing pipeline. The annual average number of human SE cases was 335.4 (sd: 154.5) over the whole study period, with the lowest number of case isolates submitted in 2020 (*n*=184) and the highest in 2023 (*n*=552). The median age of SE cases was 28 years old (IQR: 12–56 years old), and four cases had an unknown age (0.2%). There was an even distribution between sexes: 834 (50%) females, 829 (49%) males and 14 cases (0.8%) with unknown gender. The majority of SE cases were non-invasive (90%; *n*=1,514 cases), while 7% (*n*=121 cases) were invasive; in 3% (*n*=42) of SE cases, the type of infection could not be determined. Additionally, 15% (*n*=251) of SE cases were known to be travel-related. Among the phenotypic AMR profiles, the highest resistance proportion was observed for (fluor)quinolones, with 23.7% (*n*=395/1,076 isolates), followed by ampicillin (10.7%; *n*=179/1,292 isolates) and sulfamethoxazole (6%; *n*=92/1,379 isolates).

### Sequence type (ST) of isolates

Out of 1,677 SE isolates, eight (0.5%) did not meet the WGS quality criteria, leaving 1,669 isolates eligible for WGS analysis. Based on *in silico* 7-locus MLST typing, the majority of these isolates belonged to ST11 (93.9%; *n*=1,568/1,669), followed by ST183 (4.4%; *n*=74/1,669). Less common sequence types (STs) included ST1925 (0.5%; *n*=9), ST3233 (0.2%; *n*=4), ST3406 (0.1%; *n*=2), ST1971 (0.1%; *n*=1), ST4695 (0.1%; *n*=1) and ST9732 (0.1%; *n*=1). Nine isolates had an unknown MLST type (0.5%).

### Degree of clustering and population structure

**Fig. 1.**
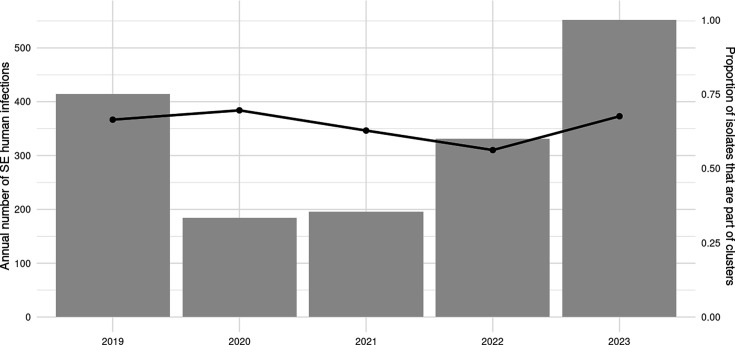
Annual incidence of *Salmonella enterica* serotype Enteritidis (SE) human infections (bar plot) and the proportion of these infections (black dot line) that are part of clusters in the Netherlands, 2019–2023, based on cgMLST using average-linkage hierarchical clustering with a cut-off of ≤3 alleles.

**Fig. 2.**
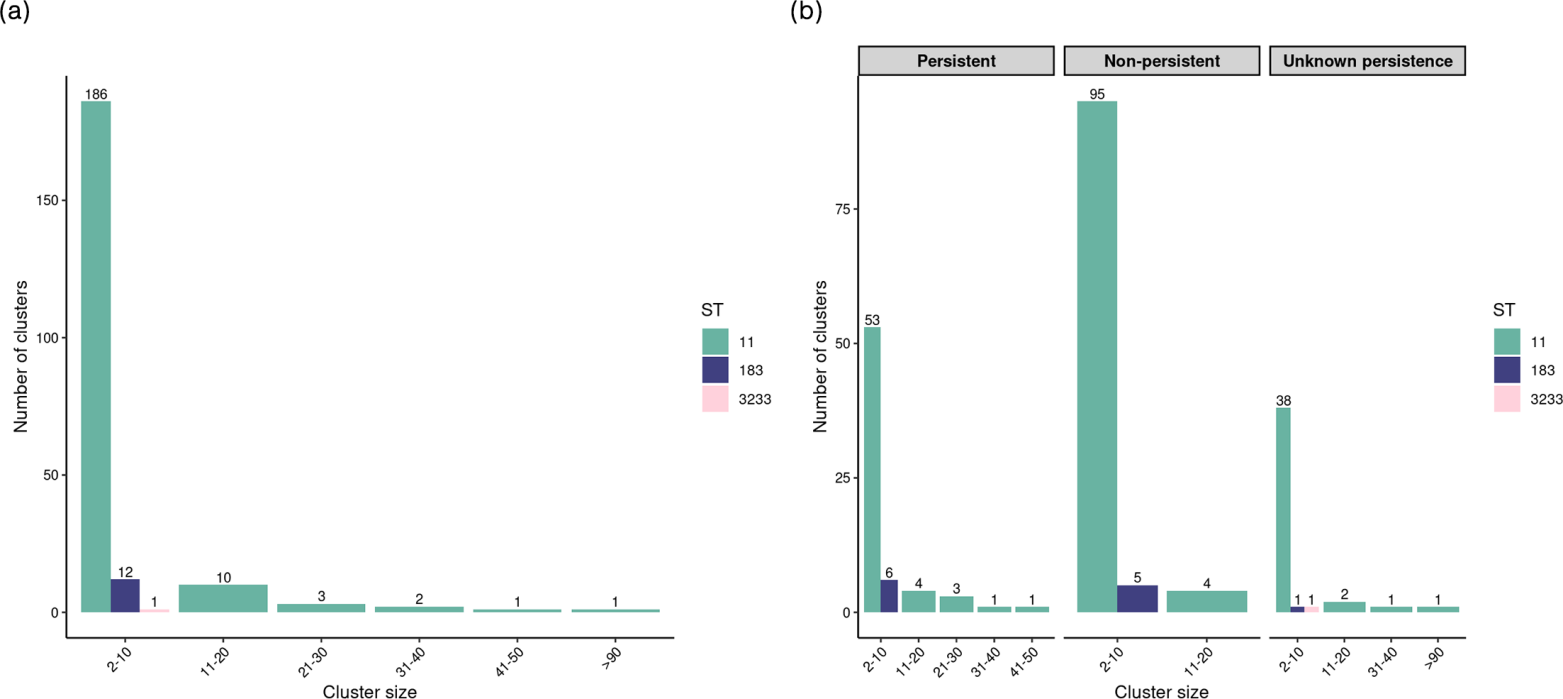
Number of clusters by cluster size (cluster size was divided into six groups according to the number of sequences), sequence type (ST) (**a**) and persistence of cluster (**b**) from human *Salmonella enterica* serotype Enteritidis sequences based on cgMLST from 2019 to 2023 in the Netherlands, using average-linkage hierarchical clustering with a cut-off of ≤3 alleles.

The cophenetic correlation exhibited high values across all three clustering methods: single-linkage (0.972), complete-linkage (0.995) and average-linkage, which showed the highest correlation with the observed distance matrix of 0.998. The optimal cluster cut-off based on Dunn2, silhouette and McClain–Rao was with *k*≤3 allelic distances (Table S3). Consequently, for cluster detection, the average linkage hierarchical clustering algorithm was used with a cluster cut-off of ≤3 alleles. This resulted in a detection of 216 clusters, comprising 1,085 (65.0%) sequences and 584 (34.9%) sequences depicted as singletons (i.e. not part of a cluster). The average size of clusters was five sequences (median=3 and range=2–94). There were 99 clusters (45.8%) containing only 2 sequences each (Table S4). Fig. 1 illustrates the annual incidence of SE human infections alongside the proportion of SE clusters over the study period. The proportion of clusters remained relatively stable, with a slight decrease in 2022, followed by a return to previous levels in 2023. Fig. 2a summarizes the total number of clusters identified by cluster size.

Among the 216 clusters identified, 79 (36.6%) included at least one known travel-related isolate. Of these, three clusters (C66, C461 and C510) were composed entirely of isolates from travel-related cases, specifically from Mauritius, Namibia and Egypt, respectively. Among all travel-related clusters, 25 clusters reported at least one case linked to travel to Turkey (*n*=41 cases). This was followed by 11 clusters that reported travel to Egypt (*n*=18 cases) and 9 clusters that reported travel to Aruba and Curaçao (*n*=15 cases) (Table S5). Of the 79 clusters, 61 (71.2%) were determined to be entirely travel-related as the total observed cases exceeded the expected number of cases, based on the probabilities of a beta-binomial distribution with parameters S_1_=1,669 and S_2_=251 (Table S6).

**Fig. 3.**
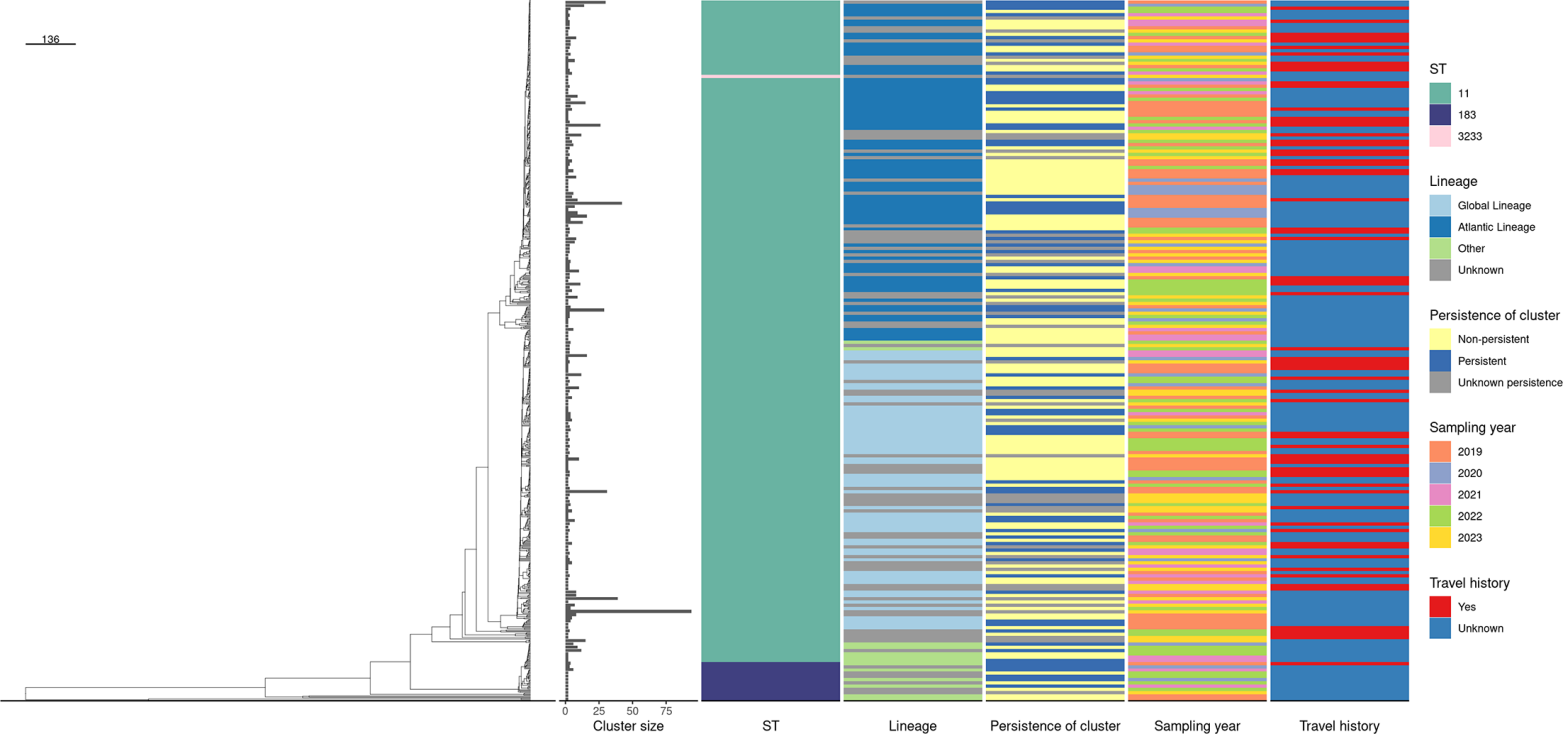
Pruned dendrogram (one sequence randomly selected from each cluster) based on average linkage hierarchical clustering of cgMLST profiles of human *Salmonella enterica* serotype Enteritidis isolates with a cut-off of ≤3 alleles. Distances between sequences were estimated using Hamming distances. The scale bar represents a Hamming distance of 136, indicating the genetic dissimilarity between clusters. For example, if a branch is half the length of the scale bar, it represents a Hamming distance of ~68. The annotation columns indicate cluster size, sequence type (ST), the estimated lineage, persistence of cluster, sampling year and travel history. For travel history, among the 79 clusters that included at least 1 travel-related case, 61 were estimated to be entirely travel-related as the number of observed travel cases exceeds the number of expected travel cases, based on the probabilities of a beta-binomial distribution with parameters S_1_=1,669 and S_2_=251.

The SE population structure based on cgMLST identified roughly three major lineages, of which the majority corresponded to the Global and Atlantic lineages (Fig. 3). The ST11 was predominant in the Global and Atlantic lineages, while the ST183 was predominant in ‘other’ lineages. Sampling year, persistence of cluster, travel history (Fig. 3), age, gender and type of infection (Fig. S1) were observed across all three lineages without structuring. Regarding phenotypic AMR, resistance to ampicillin, (fluor)quinolones and sulfamethoxazole was sporadically observed across all three lineages. However, resistance to these antimicrobials was slightly more predominant in the Global lineage (Fig. S1).

### Temporal distribution and factors associated with persistence of clusters

**Fig. 4.**
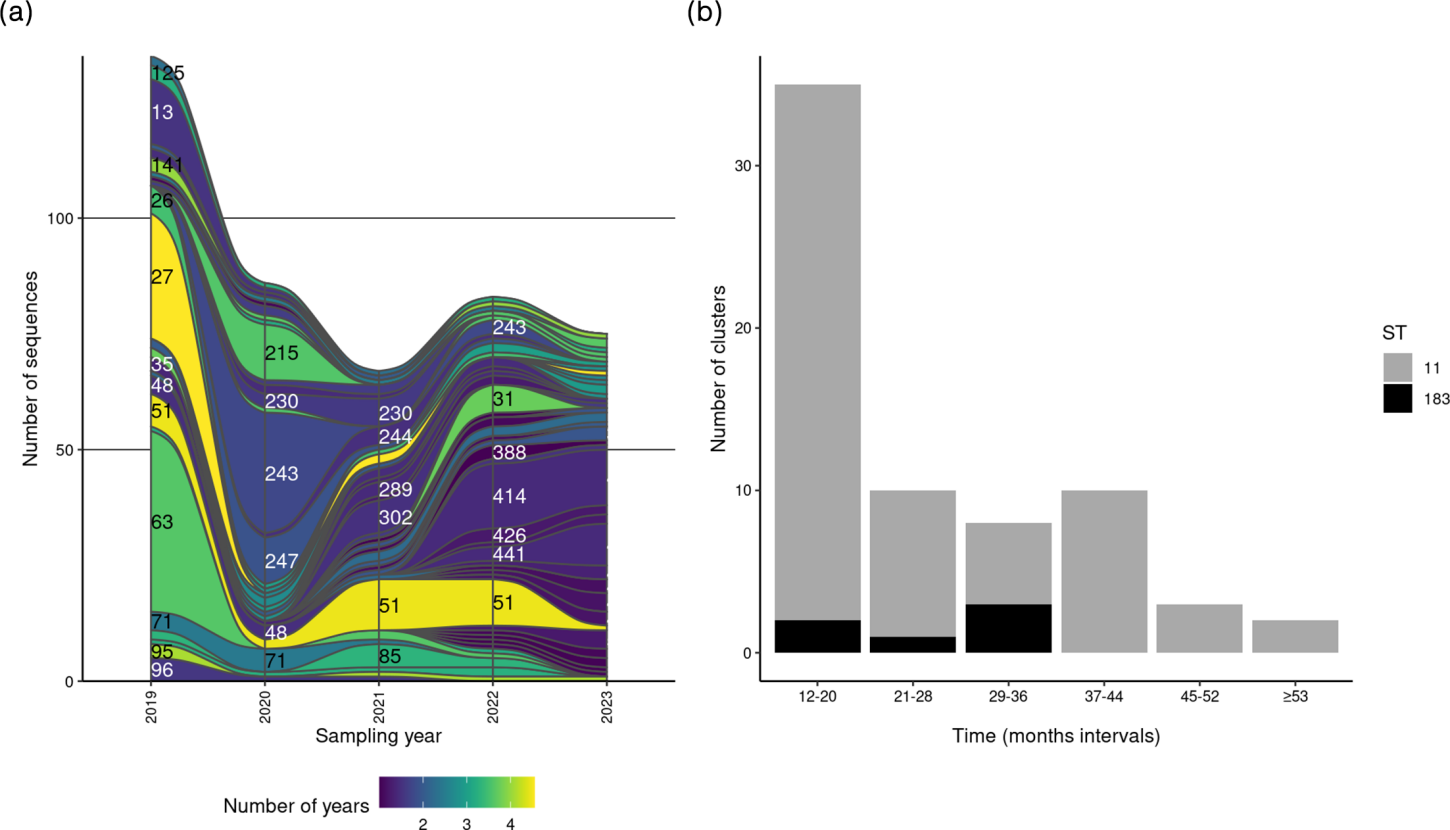
Persistent clusters across the study period, based on average-linkage hierarchical clustering with a cut-off of 3 ≤alleles: (**a**) one continuous strip represents one persistent cluster, with the corresponding cluster number indicated on the strip. The colour is indicative of the time span in which the cluster has been found. The thickness of the strip is proportional to the number of sequences in the cluster in a particular year. (**b**) The *x*-axis indicates the interval of months (six intervals) that the clusters were found by sequence type (ST); the *y*-axis indicates the number of clusters.

About one-third of all clusters (31.5%; *n*=68/216) persisted over a year, comprising a total of 446 sequences. Non-persistent clusters accounted for 104 out of 216 clusters (48.1%) and included 360 sequences. The remaining 44 clusters (20.4%) had unknown persistence and contained a total of 279 sequences (Fig. 2b). The average size of persistent clusters was higher (mean=6.6 sequences; median=4 sequences) compared to non-persistent clusters (mean=3.5 sequences; median=2 sequences). The persistent clusters had time intervals ranging from a minimum of 1 (12 months) to a maximum of 4.6 years (54 months), with a median interval of 1.6 years (18 months) (Table S7). These clusters were exclusively part of two STs: ST11 (91%; *n*=62/68) and ST183 (9%; *n*=6/68) (Fig. 2b). Persistent clusters were observed across all major lineages (Fig. 3). Fig. 4a shows the distribution of clusters that persisted over a year across the study period, with time intervals and ST depicted in Fig. 4b. Persistent SE clusters were highest in 2019, declined during 2020–2021 and then showed a slow increase towards 2023.

Results of the sensitivity analysis showed no large differences in the number of persistent clusters observed when employing the complete and single linkage clustering methods in comparison to the average-linkage clustering method. Only one additional persistent cluster was identified when using the single-linkage clustering method (Table S8). No significant factors associated with SE cases based on cluster persistence were found (Table 1).

**Table 1.** Associations of epidemiological and phenotypic AMR factors with the persistence of clusters of *Salmonella enterica* serotype Enteritidis human cases in the Netherlands, 2019–2023, based on univariable mixed-effects Cox proportional hazards model

Epidemiological and phenotypic AMR variables	Persistent*N* clusters: 68 *N* cases: 446	Non-persistent*N* clusters: 510 *N* cases: 766	Unknown-persistence*N* clusters: 222 *N* cases: 457	HRs (95% CI)**§**	*P*
Age, mean (sd), years	34.3 (25.1)	33 (25.1)	35.4 (25.4)	0.99 (0.99–1.00)	0.64
Missing (*n*)	0	3	1		
Gender, *n*					
Female	233	373	225	Ref.	
Male	208	388	228	1.02 (0.89–1.17)	0.78
Missing	5	5	4		
Type of infection, *n*					
Non-invasive	398	692	418	Ref.	
Invasive	36	51	32	0.99 (0.76–1.31)	0.99
Missing	12	23	7		
Travel history∗, *n*					
Unknown†	318	576	377	Ref.	
Yes	128	190	80	0.85 (0.60–1.19)	0.41
Ampicillin‡, *n*					
Susceptible	331	578	383	Ref.	
Resistance	50	83	46	0.84 (0.54–1.31)	0.44
Missing	65	105	28		
(Fluor)quinolones‡, *n*					
Susceptible	267	495	314	Ref.	
Resistance	114	166	115	0.87 (0.63–1.20)	0.41
Missing	65	105	28		
Sulfamethoxazole‡, *n*					
Susceptible	338	637	404	Ref.	
Resistance	43	24	25	0.61 (0.34–1.09)	0.097
Missing	65	105	28		

*Travel-related clusters were estimated to be entirely travel-related as the number of observed travel cases exceeds the number of expected travel cases, based on the probabilities of a beta-binomial distribution with parameters S_1_=1669 and S_2_=251.

†Unknown travel cases included potential domestically acquired infections and unknown travel-related infections.

‡Phenotypic antimicrobial resistance. All variables met the proportional hazard assumption. (Fluor)quinolones are collectively referred to as ciprofloxacin and nalidixic acid.

§HRs with 95% CIs. Statistically significant if *P*<0.05.

AMR, Antimicrobial resistance; HRs, Hazard ratios.

### Distribution of antimicrobial, metal and biocide resistance genes

A total of 52 unique AMR genes were identified by Resfinder, and an additional 7 AMR genes were detected by AMRfinderPlus across the whole study period. Resistance was linked with various antimicrobial classes, including aminoglycosides, beta-lactamases, sulphonamides, tetracyclines, (fluor)quinolones, trimethoprim, amphenicols, macrolides and lincosamides. Additionally, 122 isolates (7.9%) suggested multidrug resistance potential. The 10 most common AMR genes were *bla*_TEM-1B_ (beta-lactamases; 7.1%; *n*=118/1,669), *aph(6)-I*_d_ (aminoglycosides; 5.5%; *n*=91/1,669), *aph(3″)-I*_b_ (aminoglycosides; 5.5%; *n*=91/1,669), *sul2* (sulphonamides; 5.4%; *n*=90/1,669), *tet*_(A)_ (tetracyclines; 4.9%; *n*=82/1,669), *bla*_TEM-57_ (beta-lactamases; 2.1%; *n*=35/1,669), *qnrB19* (2%; quinolones; *n*=33/1,669), *bla*_TEM-135_ (beta-lactamases; 1.7%; *n*=28/1,669), *sul1* (sulphonamides; 0.5%; *n*=8/1,669) and *floR* (amphenicols; 0.5%; *n*=8/1,669). Two specific multidrug resistance transporter genes (MdsA and MdsB) were present in 99% (*n*=1,658/1,669) of all isolates. Regarding point mutations, PointFinder identified four common unique mutations, all of which are associated with (fluor)quinolones: *gyrA* p.D87Y (11.9%; *n*=198/1,669), *gyrA* p.S83Y (4.4%; *n*=75/1,669), *gyrA* p.D87N (3.7%; *n*=62/1,669) and *gyrA* p.D87G (2.6%; *n*=44/1,669). The frequency of all AMR markers identified in human SE isolates from 2019 to 2023 are shown in Table S9.

**Fig. 5.**
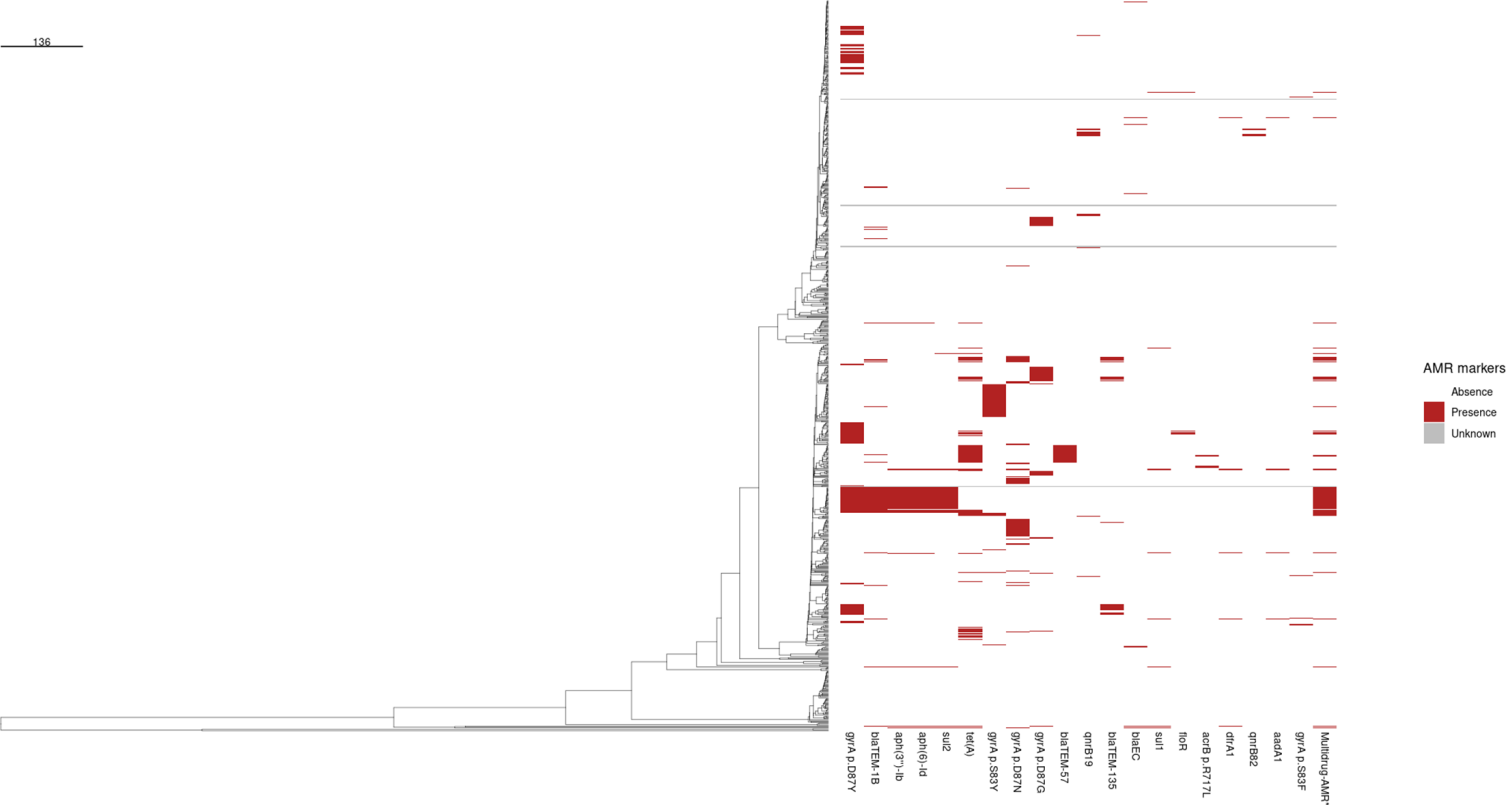
Distribution of the 20 most common antimicrobial resistance (AMR) markers and potential multidrug resistance in human *Salmonella enterica* serotype Enteritidis isolates in the Netherlands from 2019 to 2023 (ordered from most to least common). Gene presence–absence matrix showing the distribution of AMR markers across the dendrogram, which is identical to Fig. 3. The scale bar represents a Hamming distance of 136, indicating the genetic dissimilarity between clusters. For example, if a branch is half the length of the scale bar, it represents a Hamming distance of ~68. *Isolates were considered potentially multidrug-resistant if genetic AMR markers predicted resistance against ≥ 3 separate classes of antimicrobials.

Fig. 5 depicts the distribution of the 20 most common AMR markers in the dendrogram. Here, the four most common AMR markers (*gyrA* p.D87Y, *bla*_TEM-1B_, *aph(3″)-I*_b_, *aph(6)-I*_d_) were observed clustered altogether in C51, which was linked to travel to Curaçao (Global lineage). Similarly, potential multidrug-resistant isolates were observed predominantly within the same Global lineage. Other clusters with AMR markers were found scattered throughout the lineages with unknown reported travel.

Regarding markers inferring resistance to metals, AMRfinderPlus identified a total of 12 unique resistance genes, from which 99% of all isolates contained the *fief* gene (*n*=1,661/1,669), followed by the *golT* gene (99.3%; *n*=1,658/1,669) and the *golS* gene (99.3%; *n*=1,658/1,669). Less common ones (found in fewer than nine isolates) included *arsC* (0.5%; *n*=8/1,669), *arsR* (0.2%; *n*=4/1,669), *terZ* (0.1%; *n*=1/1,669), *terW* (0.1%; *n*=1/1,669), *terD* (0.1%; *n*=1/1,669), *merT* (0.1%; *n*=1/1,669), *merR* (0.1%; *n*=1/1,669), *merP* (0.1%; *n*=1/1,669) and *merC* (0.1%; *n*=1/1,669). Additionally, two genes conferring resistance to biocides were detected: *qacEdelta1* (0.4%; *n*=7/1,669) and *emrE* (0.4%; *n*=6/1,669). Except for genes being present in almost all genomes (*fief*, *golT* and *golS* genes), all other resistance genes were dispersed throughout the dendrogram without forming any distinct clusters or structures (Fig. S2).

## Discussion

Investigating frequently occurring *Salmonella* serotypes, such as SE, has historically been challenging due to the limitations of earlier, less discriminatory subtyping methods (e.g. PFGE and MLVA). However, an increasing number of countries, including the Netherlands, have incorporated WGS as a routine typing tool into their surveillance programmes for foodborne pathogens in recent years. This has significantly enhanced the ability to detect clusters and identify food products and food business operators as potential sources, thereby enabling more effective control of outbreaks both nationally and internationally. In this study, we examined the genomic epidemiology of human SE infections in the Netherlands using WGS data from isolates collected between 2019 and 2023. We analysed the temporal distribution of persistence of SE clusters and sought to identify factors that might explain the persistence of clusters. Additionally, we explored the distribution of AMR markers among human SE isolates.

### Incidence of human SE cases in the Netherlands returning to pre-COVID-19 pandemic levels

During the study period (2019 to 2023), the incidence of reported SE cases reached a historic low in 2020 due to the COVID-19 pandemic. In 2021 and 2022, the incidence increased to the pre-pandemic levels and, in 2023, was even higher than in 2019 due to the lifting of restrictions on international travel and gatherings, the reopening of (dine-in) restaurants and possibly changes in healthcare-seeking and diagnostic behaviours in the country. However, prior to the COVID-19 pandemic, the incidence of SE in human infections in the Netherlands was already increasing [3], suggesting that this increase might persist now that ‘normal’ human exposure to and reporting of SE have resumed post-pandemic. Although the underlying causes of this increase are likely to be multifactorial and interconnected, a recent study based on *Salmonella* expert elicitation in the EU identified major contributing factors throughout the food supply chain, specifically in the poultry primary production process [36]. Therefore, incorporating SE sequence data from the poultry production sector in the Netherlands and Europe may provide further insights into potential reasons for the observed increase.

### Population structure and clustering of human SE isolates from the Netherlands

The vast majority of Dutch human SE isolates, including identified clusters, belonged to ST11 (>90%), followed by ST183 (~5%). Using the average-linkage clustering method with a cut-off of three alleles distance, these isolates displayed diverse clonal structures that were consistent across different sampling years, ages, genders, travel histories and infection types, reflecting their characteristic low rate of recombination. ST11 is widely prevalent worldwide, whereas ST183 has been more recently identified in human and hedgehog populations in the UK and Western Europe, particularly affecting children [37]. The population structure of Dutch human SE isolates corresponded closely to two major and distinguishable lineages: the Global lineage (sequences from all inhabited continents) and the Atlantic lineage (mainly comprising sequences from Europe and the USA). This finding aligns with a recent study that used over 30,000 SE genomes from 98 countries worldwide [21], which showed that the population structure of SE from human, poultry and egg sources exhibited similar lineages, distinctly grouped as Global and Atlantic lineages.

### Temporal persistence of human SE clusters

About a third of the clusters were identified as persistent during the 5-year study period. Persistent clusters were generally larger in size compared to non-persistent clusters. Our study found no epidemiological or phenotypic AMR factors associated with SE cases that could explain the persistence of clusters. This can likely be explained by the limited transmission of SE between humans, suggesting the presence of persistent populations at the primary (animal) production level. Previous multi-country outbreak investigations have also noted persistent outbreak-related cases [6]. A large European outbreak in 2016 involving 14 countries linked to contaminated eggs from Polish farms saw a resurgence in cases starting late 2017 [6], continuing until 2019 [11]. Challenges continued, however, as the exact primary contamination source within the production sector remains elusive [6]. The distribution of these persistent outbreak strains may have potentially contaminated various stages of the food supply chain or originated early in the production process. A similar cluster persistence was again observed for a multi-country outbreak of SE linked to eggs from Spain that was first reported in 2019 [9]. This might have had multiple heterogeneous sources, inside or outside Spain, of which new cases are still observed to date [9].

Although non-persistent clusters constitute the majority of clusters, they are more challenging to anticipate and investigate due to their generally smaller size and more frequent occurrence. This difficulty arises from the complex epidemiological triage required to prioritize cluster investigation and the relatively low success rate of source-tracing for these smaller clusters. By the time enough epidemiological data are collected to identify a common food source, the outbreak may already be over. Thus, identifying persistent sources of SE could potentially reduce the burden of this pathogen, as ~22% of cases (two-thirds of all cases being part of clusters, with one-third of these being persistent) may be linked to persistent clusters.

### Distribution of genetic AMR markers among human SE isolates in the Netherlands

Genetic markers for AMR were detected across the entire phylogenetic tree. However, certain clusters related to AMR, particularly those exhibiting resistance to (fluor)quinolones, beta-lactamases and multidrug resistance, were more frequently observed across the Global lineage. Specifically, mutations such as *gyrA* p.D87Y and genes like *bla*_TEM-1B_, *aph(3″)I*_b_, and *aph(6)-I*_d_, including multi-drug resistant markers, were predominantly present in cases linked to travel to Curaçao. These observations are consistent with those of previous studies that reported similar resistance mechanisms among SE genomes worldwide [38,40]. While it remains unknown whether these resistance mechanisms would confer phenotypic AMR among the Dutch SE isolates, the prevalence of phenotypic resistance to (fluor)quinolones, ampicillin, sulfamethoxazole and tetracycline among SE isolates in the Netherlands increased steadily until 2019 [13].

Multidrug-resistant non-typhoidal *Salmonella* strains have been associated with more severe clinical outcomes, including invasive infections [41,42]. In high-income countries, including the Netherlands, SE typically causes self-limited gastroenteritis, a non-invasive form. However, SE is also responsible for slightly more than a quarter of invasive infections, a form where the pathogen is disseminated through the bloodstream to otherwise sterile organs [2]. These invasive infections generally display lower levels of (multi) antimicrobial resistance [2]. In our study, the majority of human SE infections were non-invasive (90%), and overall, the phenotypic AMR levels were lower (>24%). Furthermore, our results of WGS revealed no distinct phenotypic AMR patterns that differentiate between invasive and non-invasive within the Dutch SE population structure. Conversely, in lower income regions, such as sub-Saharan Africa, SE is a major cause of invasive infections with multi-drug resistance [43]. This discrepancy may be attributed to regional variations in AMR and disease severity. A recent study employing WGS on 675 SE isolates from 45 countries identified two novel clades that are geographically confined to specific regions in Africa. These clades demonstrated genomic degradation and a unique prophage repertoire and contained an expanded multidrug resistance plasmid, suggesting distinct evolutionary adaptations [44]. Therefore, future research that explores the comparative genomics of invasive and non-invasive SE strains could provide valuable insights into changes in population genetics and the underlying mechanisms of pathogenicity.

### Strengths and limitations

This is the first study to investigate the persistence of human SE clusters in the Netherlands, using WGS data for all reported human SE cases. However, several limitations of this study should be acknowledged. Firstly, the surveillance system for human salmonellosis captures an estimated 60% of all laboratory-confirmed salmonellosis cases in the country [14], which may lead to an underestimation of the size and persistence of clusters. Secondly, data on travel history are often missing, potentially resulting in an underestimation. To mitigate this issue, we assessed whether the observed number of cases within each cluster was higher than expected. If so, the whole cluster was considered travel-related. Nonetheless, we cannot exclude the possibility of misclassifying clusters as either travel or non-travel-related. Finally, in our study, we observed that the persistent SE clusters declined starting in 2020 and began to increase slowly towards the end of 2021. This trend coincided with the implementation of social distancing measures during the COVID-19 pandemic, such as restrictions on social gatherings and the closure of restaurants and festivals (where food is often served), as well as restrictions on international travel. These measures significantly reduced human salmonellosis cases, particularly affecting the epidemiology of SE human cases in the Netherlands [45]. Since WGS became a routine typing method for SE surveillance in the Netherlands in 2019, we analysed data from 2019 onwards. The lack of a significant association between the factors assessed and the persistence of SE clusters may be partially influenced by the impact of COVID-19 measures, which we were unable to adjust for in this analysis.

### Recommendations

Our study showed that a cut-off of ≤3 alleles was optimal for clustering of SE isolates using cgMLST, as supported by three indices: Dunn2, silhouette and McClain–Rao. Given the clonal nature of SE, using more stringent allele thresholds for genomic cluster identification along with robust epidemiological data, particularly during outbreak, investigations might be recommended. A previous study in the USA [46] suggested employing a lower allele threshold for genomic clustering, leading to enhanced cluster resolution and more refined cluster identification. Moreover, to better understand the biological or ecological nature of persistent clusters, we recommend conducting further comparative genomics, phylogenetic and genome-wide association studies (GWAS) with higher resolution than cgMLST, including SE isolates from food samples. Recent advancements in hierarchical machine learning models have enabled more precise geographical source predictions from SE genome sequences [47]. Therefore, we recommend further exploration of the relationship between the persistence of SE clusters and genetic signatures. For the detection of SE clusters using WGS, we recommend exploring alternative methods that have recently shown better effectiveness and speed for the detection of SE outbreaks, such as the multilevel genome typing (MGT) scheme. This method offers varying levels of resolution for identifying specific genetic types. Specifically, the serovar cgMLST level MGT9 may be useful for national or international outbreak investigations [48]. Additionally, since we were unable to account for the impact of the COVID-19 pandemic measures on the association between epidemiological factors and cluster persistence, we suggest further exploration of these relationships in a post-COVID-19 period. Finally, it may be worthwhile to investigate whether a cut-off of 3 alleles could offer improved resolution compared to the currently used 5-allele cut-off in European SE outbreak investigations.

In conclusion, our results offer deeper insights into the population structure of SE isolates from human infections in the Netherlands. Notably, approximately one-third of SE clusters persisted for over a year without any identifiable factor explaining this persistence. Factors related to the primary production of poultry might provide more insights into this persistence. We recommend conducting additional comparative genomic and phylogenetic studies, as well as GWAS, using higher-resolution methods than cgMLST in a post-COVID-19 period. Including representative collections of SE isolates from food samples will also help investigate factors associated with persistence clusters.

## Supplementary material

10.1099/mgen.0.001394 Uncited Supplementary Material 1.

10.1099/mgen.0.001394 Uncited Supplementary Material 2.

## Abbreviations

AMR: antimicrobial resistance
AST: antimicrobial susceptibility testing
CDF: cumulative beta binomial distribution function
cgMLST: core-genome multi-locus sequence typing
CIs: confidence intervals
COVID-19: coronavirus disease of 2019
EU: European Union
EUCAST: European Committee on Antimicrobial Susceptibility Testing
GWAS: genome-wide association studies
HRs: hazard ratios
IQR: interquartile range
MGT: multilevel genome typing
MIC: minimum inhibitory concentration
MLST: multilocus sequence typing
MLVA: multiple-locus variable-number tandem repeat analysis
PFGE: pulsed-field gel electrophoresis
RIVM: The Dutch National Institute for Public Health and the Environment
SD: standard deviation
SE: *Salmonella enterica* serotype enteritidis
ST: sequence type
WGS: whole-genome sequencing
