# Supplementary material for: Genomic epidemiology of Salmonella Enteritidis human infections in the Netherlands, 2019 to 2023
Source: Microb Genom. 2025 Apr 23;11(4):001394. doi: 10.1099/mgen.0.001394 (PMC12044193; doi:10.1099/mgen.0.001394)

R code for the analysis of factors associated with persistence of clusters

Univariable mixed-effect Cox proportional hazard regression models

```
age.m <- coxme(Surv(time, status) ~ age + (1|s_3), data = df_epidata)
summary(age..m)
exp(confint(age.m)) # Obtaining the 95%CI of the hazard ratios (HRs).
cox.zph(age.m) # Checking the proportional hazard assumption


gender.m <- coxme(Surv(time, status) ~ gender + (1|s_3), data = df_epidata)
summary(gender.m)
exp(confint(gender.m))
cox.zph(gender.m)


travel.m <- coxme(Surv(time, status) ~ travel + (1|s_3), data = df_epidata)
summary(travel.m)
exp(confint(travel.m))
cox.zph(travel.m)


inf.m <- coxme(Surv(time, status) ~ infection_type + (1|s_3), data = df_epidata)
summary(inf.m) # Significant
exp(confint(inf.m))
cox.zph(inf.m)


amp.m <- coxme(Surv(time, status) ~ amp + (1|s_3), data = df_epidata)
summary(amp.m) #Non-significant
exp(confint(amp.m))
cox.zph(amp.m)


fluor.m <- coxme(Surv(time, status) ~ fluor + (1|s_3), data = df_epidata)
summary(fluor.m) #Non-signficant
exp(confint(fluor.m))
cox.zph(fluor.m)


smx.m <- coxme(Surv(time, status) ~ smx + (1|s_3), data = df_epidata)
summary(smx.m) #Non-signficant
exp(confint(smx.m))
cox.zph(smx.m)
```

*Since none of these factors were significant, no multivariable model was built.

Supplementary Figure 1. Pruned dendrogram (one sequence randomly selected from each cluster) based on average linkage hierarchical clustering of cgMLST profiles of human *Salmonella* Enteritidis infections with a cut-off of ≤3 alleles. Distances between sequences were estimated using Hamming distances. The scale bar represents a Hamming distance of 136, indicating the genetic dissimilarity between clusters. For example, if a branch is half the length of the scale bar, it represents a Hamming distance of approximately 68. The annotation columns indicate lineage, age group, gender, type of infection and phenotypic AMR (ampicillin, fluoroquinolones, and sulfamethoxazole). Fluoroquinolones are collectively referred to Ciprofloxacin and Nalidixic acid in this figure.

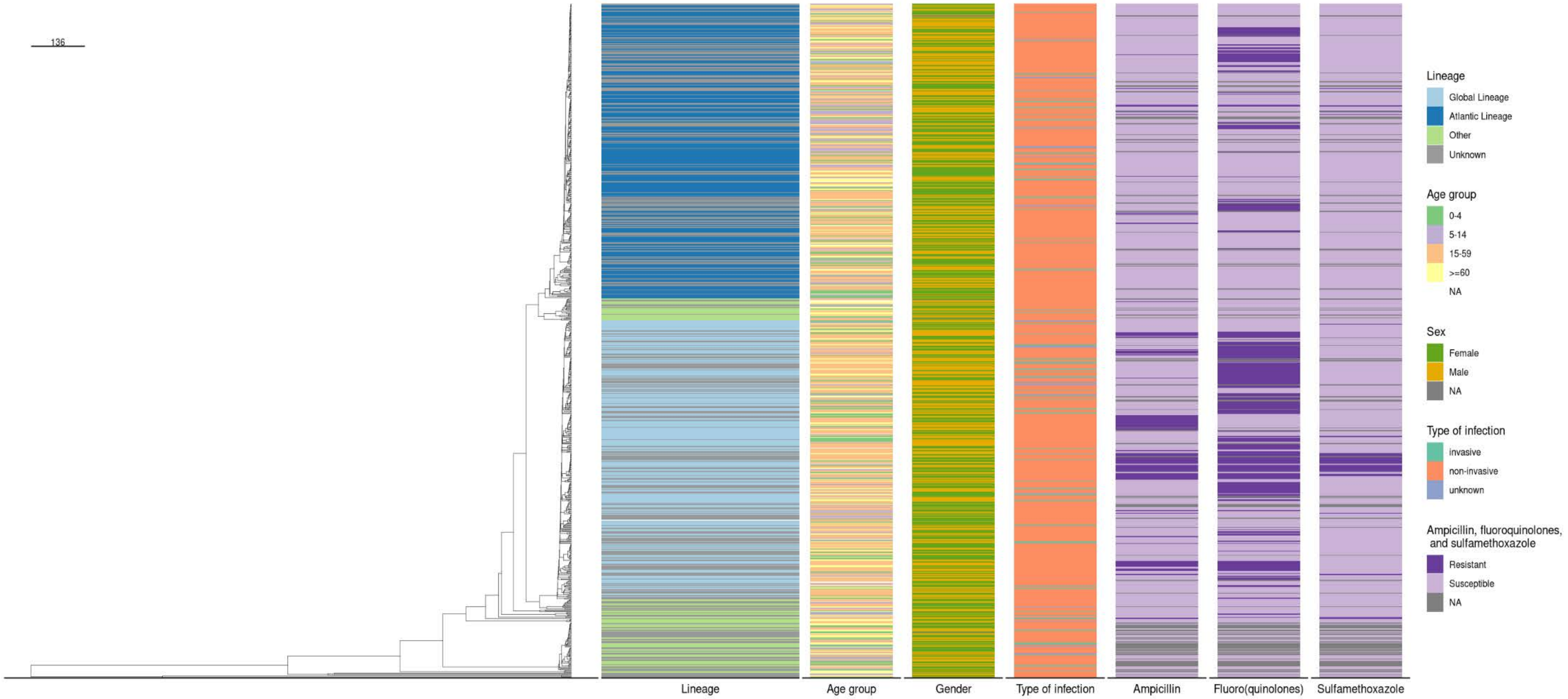

Supplementary Figure 2. Distribution of metal and biocides resistance markers from *Salmonella* Enteritidis human infections in the Netherlands from 2019 to 2023. Gene presence-absence matrix showing the distribution of metal and biocides resistance genes across the pruned dendrogram (dendrogram is identical to **Supplementary Figure 1**). The scale bar represents a Hamming distance of 136, indicating the genetic dissimilarity between clusters. For example, if a branch is half the length of the scale bar, it represents a Hamming distance of approximately 68.

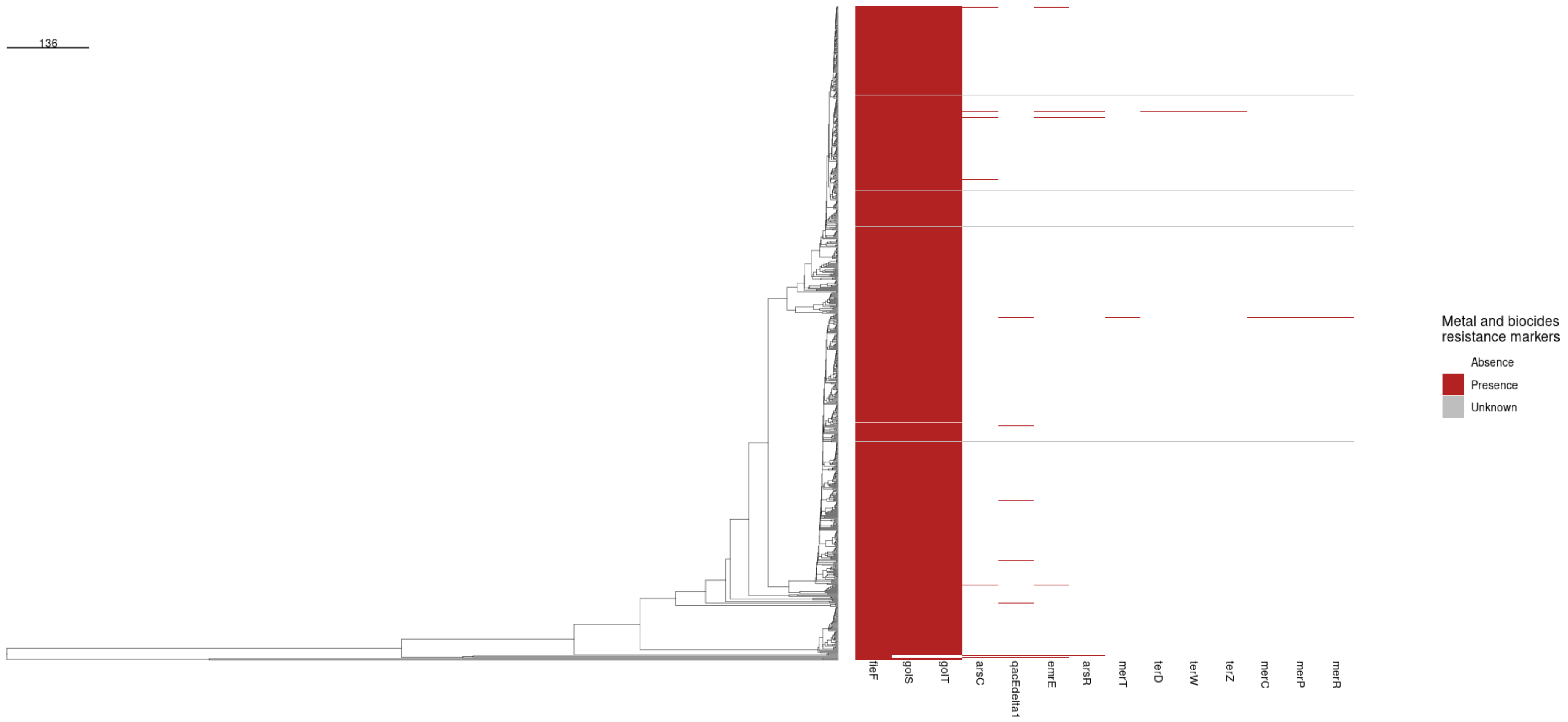

Supplement: Uncited Supplementary Material 1. [file mgen-11-01394-s001.pdf]
